# PI3K/Akt promotes feedforward mTORC2 activation through IKKα

**DOI:** 10.18632/oncotarget.8383

**Published:** 2016-03-25

**Authors:** Han C. Dan, Ricardo J. Antonia, Albert S. Baldwin

**Affiliations:** ^1^ The Lineberger Comprehensive Cancer Center, University of North Carolina School of Medicine, Chapel Hill, NC, USA; ^2^ Marlene and Stewart Greenebaum Cancer Center, University of Maryland School of Medicine, Baltimore, MD, USA; ^3^ Department of Pathology, University of Maryland School of Medicine, Baltimore, MD, USA

**Keywords:** IKKα, Akt, mTOR, Rictor, Sin1

## Abstract

The ser-thr Akt plays a critical role in the regulation of cell survival, cell growth and proliferation, as well as energy metabolism and is dysregulated in many cancers. The regulation of Akt activity depends on the phosphorylation at two sites: (i) Thr308 in the activation loop by phosphoinositide-dependent kinase-1 (PDK1) and (ii) Ser473 hydrophobic motif at the carboxyl terminus by a second activity termed PDK2, which is the mTORC2 complex composed of mTOR, rictor, and Sin1. Previously we demonstrated that IKKα, a component of the IKK complex that controls NF-κB activation, participates in the Akt-dependent regulation of mTORC1. Here we have explored a potential involvement of IKKα in controlling Akt activity and whether this may involve mTORC2. The experiments show that IKKα associates with mTORC2 in several cancer cells in a manner dependent on PI3K/Akt activity and that IKKα positively promotes Akt phosphorylation at Ser473 and at Thr308. Moreover, IKKα enhances mTORC2 kinase activity directed to Akt on Ser473 and Akt-mediated phosphorylation of FOXO3a and GSK3β, but not other Akt-associated targets such as TSC2 and PRAS40, indicating the existence of multiple mechanisms of Akt activation in cells. In addition, loss of IKKα suppresses growth factor-induced Akt activation associated with mTORC1 inhibition. These results indicate that IKKα serves as a feedforward regulator of mTORC2 and that IKKα could serve as a key therapeutic target to block mTORC2 and Akt activation in some cancers.

## INTRODUCTION

Akt family proteins are an evolutionarily conserved group of serine-threonine kinases which phosphorylate key substrate involved in numerous cellular pathways [1, 2]. The initiating step in Akt activation is the binding of PIP3 to the pleckstrin homology (PH) domain of Akt, and subsequent translocation of Akt to the plasma membrane, where it is activated by phosphorylation through PDK1 [1 -3] and by PDK2 which has been shown to be the mTOR complex 2 (mTORC2) [4]. In cancers, Akt is often found constitutively activated downstream of growth factor receptor signaling, through activating mutations in PI3K, or following PTEN loss of expression or mutation [3, 5, 6]. Activated Akt promotes cell survival, cell growth and proliferation, and energy metabolism in human cancers [3, 5]. Understanding the control of Akt is critical to provide insights into a variety of physiological and cancer-associated mechanisms.

The mammalian target of rapamycin (mTOR) is a conserved protein kinase that exists in two distinct complexes, mTORC1 and mTORC2 [2, 7]. mTORC1, which contains mTOR, Raptor and GβL, controls cell growth, at least partly, through its ability to phosphorylate S6K and 4EBP1, key regulators of mRNA translation [2, 7, 8]. mTORC1 can be activated downstream of signaling induced by growth factors, such as IGF-1 and insulin, in a manner controlled downstream of PI3K/Akt [7, 8]. It has been shown that active Akt activates mTORC1 through phosphorylation of the tumor suppressor TSC2 to release TSC2 inhibition of the GTPase Rheb leading to positive regulation of mTORC1 [7 - 11]. Akt has also been shown to phosphorylate PRAS40 to lead to mTORC1 activation [12, 13]. mTORC2, which contains mTOR, Rictor, Sin1 and GβL, is rapamycin-insensitive and has been reported to regulate the actin cytoskeleton by modulating protein kinase C and Rho-family small GTPases [2, 4, 8]. As mentioned above, mTORC2 has also been shown to function as the PDK2 activity to phosphorylate Akt on ser473 [4]. Mechanisms for inducible control of mTORC2 activity have been elusive, but it was shown recently that PIP3, generated downstream of PI3K, binds to Sin1 to promote mTORC2 activity [14]. A separate study indicated that Sin1 acetylation is important for mTORC2 activation [15].

The transcription factor nuclear factor kappa B (NF-κB) pathway is activated downstream of a variety of inflammatory mediators and growth factor pathways [16, 17]. Cytokine and growth factor-induced activation of the IκB kinase (IKK) complex (IKK) is a key step involved in NF-κB activation leading to degradation of the inhibitor IκB and to the ability of NF-κB to accumulate in the nucleus and and initiate transcription of downstream target genes. The canonical NF-κB signaling pathway activates a heterodimer of a RelA-p50 complex and utilizes an IKK complex that is comprised of two catalytic subunits, IKKα and IKKβ, in association with a regulatory subunit IKKγ [16]. In the non-canonical signaling pathway, a dimer of IKKα promotes the activation of a p52-RelB heterodimer [16]. IKK and NF-κB activity are linked with cancer progression through the control of survival, cell proliferation, and angiogenesis [17-19]. We recently reported that IKKα associates with mTORC1 in PTEN-deficient cancer cells in an Akt-dependent manner to regulate mTOR kinase activity directed to S6K and 4E-BP1 [20-22]. In this pathway, IKKα phosphorylates mTOR in the mTORC1 complex to promote its kinase activity [23]. Reciprocally, Akt-induced association between mTOR/Raptor and IKK leads to the activation of NF-κB activity [22]. Recently it was reported that IKKα and IKKβ associate with the mTORC2 complex and that knockdown of either IKKα or IKKβ reduced Akt S473 phosphorylation [24]. In the present study, we investigated the potential molecular link between IKK and mTORC2. We found that IKKα interacts with the mTORC2 complex in several cancer cells to control mTORC2 kinase activity directed to Akt Ser473. IKKβ was found not to be critical for this mechanism in a variety of cancer cells. Interestingly, PI3K/Akt signaling promotes an interaction between IKKα and mTORC2, driving mTORC2 activity. Loss of IKKα parallels loss of mTORC2 activity in affecting a subset of Akt-targeted substrates. The data indicate that IKKα functions in a feedforward pathway downstream of PI3K/Akt to promote mTORC2-mediated Akt activation.

## RESULTS

### Depletion of IKKα impairs Akt phosphorylation in mammalian cancer cells

**Figure 1:**
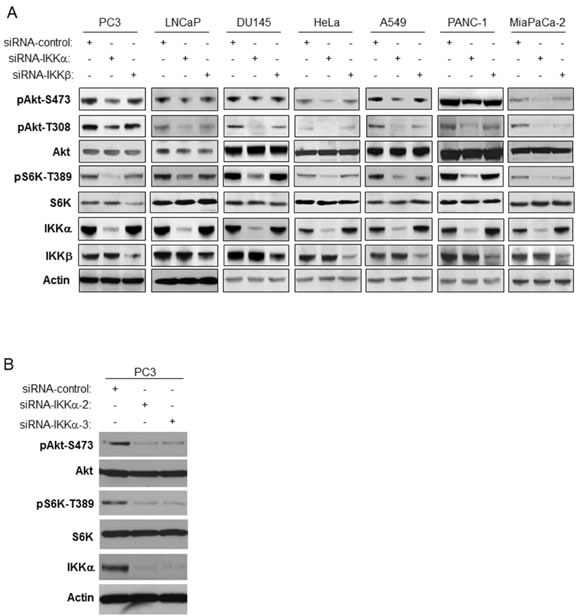
Knockdown of IKKα decreases both Akt phosphorylation and mTORC1 activity **A.** PC3, LNCaP, DU145, HeLa, A549, PANC-1 and MiaPaCa-2 cells were transfected with control siRNA, and siRNAs to IKKα and IKKβ as indicated. The cells were lysed 48 hrs after transfection and the levels of IKKα, IKKβ, and β-actin and endogenous phosphorylation of S6K and Akt were determined by immunoblotting with the indicated antibodies. The experiments were carried out on three separate occasions. **B.** PC3 cells were transfected with siRNA control, siRNA-IKKα-2, or siRNA-IKKα-3, and lysed 48 hrs after transfection. Levels of the indicated proteins or phospho-proteins were detected by antibodies used in Figure 1A.

Our previous studies demonstrated that IKKα plays an important role in mTORC1 activation downstream from Akt [20-23). Those results prompted us to investigate a potential involvement of IKKα in the regulation of Akt activity. To address this, we first performed RNAi experiment in two prostate cancer cell lines, PC3 and LNCaP, in which we previously demonstrated that IKKα has a positive function on mTORC1. It should be noted that PC3 cells do not express PTEN while LNCaP cells express a mutated form of PTEN. As shown in Figure 1A, siRNA specific for IKKα and IKKβ was effective at reducing the expression levels of these kinases. Subsequently, Akt activity was assayed using phosphospecific antibodies. The results revealed that knockdown of IKKα suppressed phosphorylation of Akt at both ser473 and thr308 (Figure 1A), which are important phosphorylation residues required for full Akt activation. Additionally, as expected [20] the knockdown of IKKα in PC3 cells also blocked phosphorylation of S6K at thr389, a marker for mTORC1 activity. There was no effect on the levels of endogenous Akt and S6K under these experimental conditions (Figure 1A). Interestingly, knockdown of IKKβ displayed a minimal effect on phosphorylation of Akt and S6K, which contrasts with the findings of Xu et al [24]. These data indicated that IKKα positively modulates the activity of both mTORC1 and Akt in PTEN deficient prostate cancer cells (and see overall signaling pathways outlined in Figure 8). Next, we conducted RNAi experiment in both HeLa cells and DU145 prostate cancer cells, both of which are PTEN wild type, and found that knockdown of IKKα also markedly decreased phosphorylation of both Akt and S6K (Figure 1A). We then studied whether IKKα promotes Akt and mTOR activity in other mammalian cancer cell lines. We knocked down IKKα and IKKβ in A549, PANC1 and MiaPaCa-2 cells and measured the activity of Akt and mTOR. The results indicate that the phosphorylation of both Akt and S6K was dramatically reduced after the knockdown of IKKα in these cells. IKKβ knockdown slightly decreases the phosphorylation of Akt and mTOR (Figure 1A). Identical results were obtained using distinct IKKα siRNA pools (Figure 1B). These results indicate that IKKα plays an important role in the activation of both Akt and mTORC1 independent of PTEN loss.

### Overexpression of IKKα induces Akt activation

**Figure 2:**
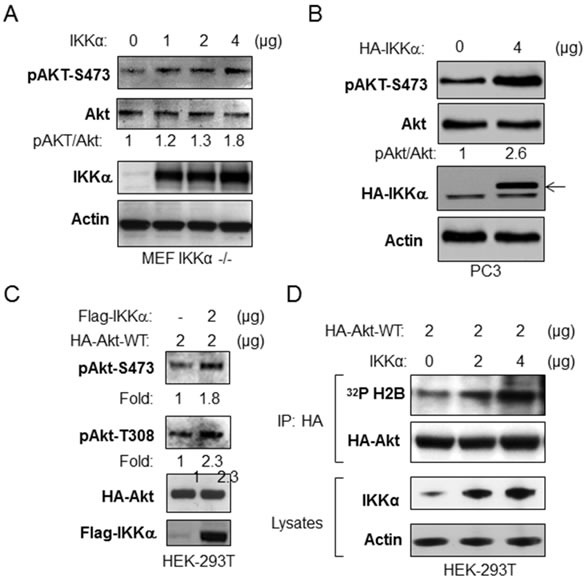
Overexpression of IKKα increases phosphorylation and kinase activity of Akt **A.** IKKα−/− MEF cells were transfected with different doses of IKKα as indicated, and the levels of phosphorylation of Akt, phospho-Akt, flag-IKKα and β-actin were measured by immunoblotting. **B.** PC3 cells were transfected with HA-IKKα and the levels of phosphorylation of Akt and levels of Akt, HA-IKKα and β-actin were detected. Results are representative out of at least 3 experimental repetitions. **C.** HEK293T cells were co-transfected with HA-Akt-wild type and flag-IKKα-wild type as indicated, and the levels of phosphorylation of Akt, HA-Akt, flag-IKKα and β-actin were detected. Results are representative out of at least 3 experimental repetitions. **D.** Expression of IKKα enhances *in vitro* Akt kinase activity. HEK293T cells were co-transfected with different amounts of IKKα and with HA-Akt. The kinase activity of the HA-Akt immunoprecipitate to histone H2B was determined. The experiments were repeated three times.

In order to extend the results above, we asked if IKKα expression would induce Akt activity. We first transfected HA-tagged wild type IKKα into IKKα null MEFs and measured phosphorylation of endogenous Akt. As shown in Figure 2A, transfection of IKKα leads to expression of IKKα, which was accompanied by an increase of phosphorylation of Akt at serine 473. To test if IKKα activates Akt in cancer cells, IKKα was transfected into PC3 cells and the phosphorylation of endogenous Akt was determined. The data demonstrate that expression of IKKα leads to an increase of phosphorylation of Akt (Figure 2B) similar to the data generated using IKKα null MEF cells. Next, we tested whether overexpression of IKKα induces phosphorylation of exogenously expressed Akt. HEK 293T cells were co-transfected as indicated with a Flag-tagged wild type IKKα and HA-tagged wild type Akt. Cells were lysed and immunoprecipitations of cellular lysates were performed with the anti-HA antibody. As shown in Figure 2C, IKKα expression increases exogenous Akt phosphorylation at both serine 473 and threonine 308. To determine whether IKKα promotes Akt kinase activity, we co-transfected IKKα with HA-tagged Akt and then immunoprecipitated Akt from the cell lysates with the HA antibody, which was used for an *in vitro* Akt kinase assay against histone H2B, a classic Akt substrate. Our results indicate that overexpression of IKKα significantly increased Akt kinase activity (Figure 2D). Overall, these results demonstrate that IKKα induces Akt phosphorylation and kinase activity.

### IKKα-driven Akt activity does not affect Akt phosphorylation of TSC2 and PRAS40

**Figure 3:**
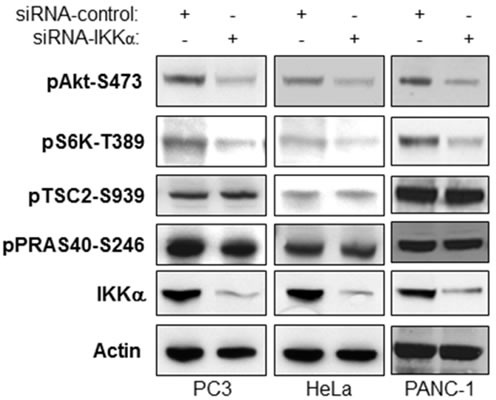
IKKα-mediated Akt activity does not affect Akt-directed phosphorylation of TSC2 and PRAS40 PC3, PANC-1 and HeLa cells were transfected with control siRNA or siRNAs to IKKα as indicated. The cells were lysed 48 hrs after transfection and the levels of IKKα and β-actin and endogenous phosphorylation of Akt, S6K, TSC2, and PRAS40 and were determined by immunoblotting with the indicated antibodies.

Downstream of Akt signaling, IKKα positively regulates mTORC1 activity to modulate S6K and 4E-BP1 phosphorylation [20-23]. It has been shown that Akt activates mTORC1 through inhibition of the TSC1/TSC2 complex by TSC2 phosphorylation. Another recently reported intermediary for Akt activation of mTORC1 is PRAS40 which normally inhibits mTORC1 but is inhibited by Akt through phosphorylation to promote mTORC1 activity. Here, we tested if IKKα-mediated Akt activation affects Akt-dependent phosphorylation of TSC2 and PRAS40, and therefore whether one effect of IKKα to promote mTORC1 is through the control of Akt. We knocked down IKKα in PC3 (PTEN null and high Akt Activity), PANC-1 (high Akt activity) and HeLa (lower Akt activity) cells and tested the effects on phosphorylation of Akt, TSC2 and PRAS40 as well as mTORC1 activity. Our results indicate that loss of IKKα leads to a decrease of Akt activity (Figure 3), as shown by loss of pAkt-S473, which is consistent with results shown in Figures 1 and 2. However, the reduction of phosphorylation of TSC2 and PRAS40 at published Akt sites is not observed with loss of IKKα while loss of mTORC1 is observed (loss of phosphorylation of S6K) as expected from our previous work [20]. We therefore conclude that IKKα-mediated mTORC1 activation is TSC2 and PRAS40-independent and that phosphorylation of Akt at S473 does not correlate with phosphorylation of PRAS40 and TSC2 (and see below for further discussion).

### IKKα inhibition blocks stimulation of Akt activity induced by mTORC1 inhibition

**Figure 4:**
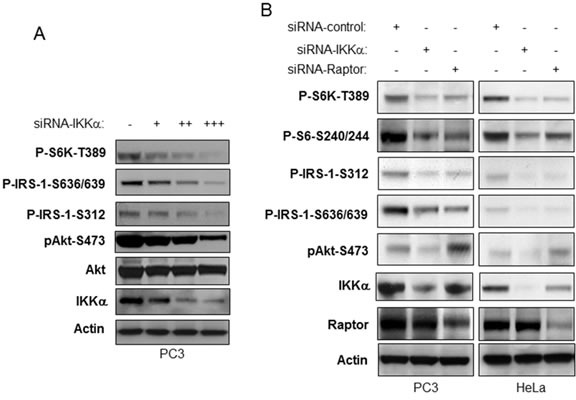
IKKα is required for activation of Akt downstream of mTORC1 inhibition **A.** PC3 cells were transfected with different amounts of siRNA IKKα. The levels of endogenous phosphorylation of S6K, IRS-1, and Akt as well as levels of IKKα, mTOR and β-actin were determined by immunoblotting with the indicated antibodies. **B.** PC3 and HeLa cells were transfected with control siRNA or siRNA to IKKα and Raptor as indicated. The cells were lysed 48 hrs after transfection and the levels of IKKα, Akt, Raptor and β-actin and of endogenous phosphorylation of S6K, IRS-1 and Akt were determined by immunoblotting with the indicated antibodies.

It has been shown that mTORC1 and its downstream effecter S6K negatively regulate Akt activity through serine phosphorylation of insulin receptor substrate-1 (IRS-1) [reviewed in 25]. Thus therapeutics that block mTORC1 have been shown to be less effective because of subsequent upregulation of Akt activity. We investigated if loss of IKKα affects IRS-1 phosphorylation and whether Akt would correspondingly be activated. We transfected different doses of siRNA IKKα into PC3 cells to decrease endogenous IKKα expression levels and tested this effect on IRS-1 phosphorylation. Our data demonstrate that knockdown of IKKα significantly decreases phosphorylation of IRS-1 at both serine 636/639 and serine 312 consistent with loss of mTORC1 and S6K activity (Figure 4A). Interestingly, loss of IKKα, while blocking mTORC1, repressed Akt activation. We compared the differences between loss of IKKα and Raptor, a key component of mTORC1, in their influence on phosphorylation of S6K, S6, IRS-1 and Akt. As shown in Figure 4B, siRNA to IKKα and to Raptor shows similar decreases of S6K, S6 and IRS-1 phosphorylation through mTORC1. However, knockdown of IKKα led to decreased Akt phosphorylation but knockdown of Raptor caused an increase in Akt phosphorylation, consistent with the known inhibitory action of mTORC1/S6K on IRS-1 activity [25]. These data are consistent with a key role for IKKα in promoting Akt activation.

### IKKα interacts with both mTORC1 and mTORC2 downstream of PI3K signaling

**Figure 5:**
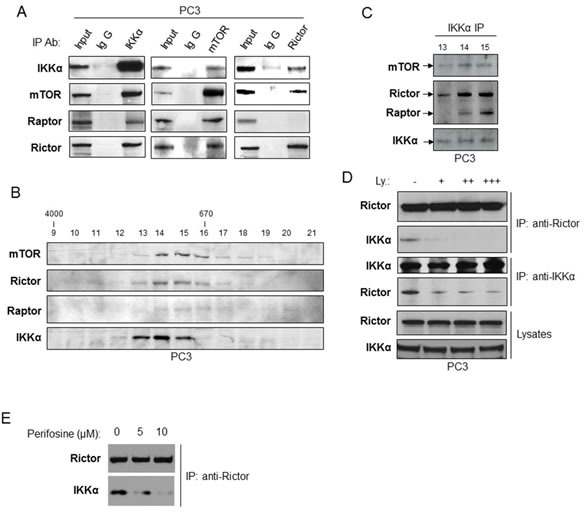
IKKα associates with both mTORC1 and mTORC2 Association with mTORC2 is dependent on PI3K and Akt. **A.** Immunoprecipitations of endogenous IKKα (left panel), mTOR (middle panel) or Rictor (right panel) prepared from PC3 cells lysates which were analyzed for IKKα, mTOR, Rictor or Raptor levels using indicated antibodies. Mouse (left panel) or rabbit (middle and right panels) IgG were used as negative control, and total cell lysate (1% input) indicates expression of mTOR complex proteins. **B.** and **C.** PC3 cell lysates were separated on a Superose 6 10/300 GL column. The indicated fraction numbers (fractionation 9-21) and their mTOR immunoprecipitates (fractionation 13-15) were analyzed by immunoblotting with the indicated antibodies. **D.** PC3 cells were treated with different doses of the PI3K inhibitor LY294002 for one hour, and the cell lysates were immunoprecipitated with Rictor or IKKα antibody, and association was determined by immunoblotting with the indicated antibodies. **E.** PC3 were treated with the Akt inhibitor Periforsine (KRX-0401) for one hour and the interaction between IKKα and the mTORC2 complex was measured as described in part D.

We previously reported that IKKα interacts with and activates mTORC1 [20] and data described above demonstrate that IKKα affects Akt phosphorylation. Given the critical role of the mTORC2 complex in activation of Akt, we thus determined whether IKKα also associates with the mTORC2 complex. To test this hypothesis, co-immunoprecipitation experiments were carried out in PC3 cells. First, cell lysates from PC3 cells were immunoprecipitated with the IKKα antibody and probed with antibodies for mTOR, Raptor and Rictor following gel electrophoresis. As shown in Figure 5A (left panel), immunoprecipitation with the IKKα antibody revealed robust association with mTOR, Raptor (part of the mTORC1 complex) and Rictor (part of the mTORC2 complex). Similarly, immunoprecipitation with the mTOR antibody demonstrates mTOR association with IKKα, Raptor and Rictor (Figure 5A, middle panel). Importantly, immunoprecipitation with the Rictor antibody showed Rictor interaction with IKKα and mTOR (Figure 5A, right panel). These data demonstrate that IKKα is associated with both mTORC1 and mTORC2 in PC3 cells. Our previous size fractionation experiments [23] demonstrated that IKKα co-fractionates with mTORC1 in PC3 cells, so we conducted similar experiments to test whether IKKα also co-fractionates with the mTORC2 complex. PC3 cell extracts were analyzed by Superose 6 HPLC column chromatography, and column fractions 9 through 21 were analyzed by immunoblotting using antibodies for mTOR, Raptor, Rictor, and IKKα (Figure 5B). As shown in Figure 5B, mTOR, Rictor and Raptor were found at the highest levels in fractions 14-16, with lower levels in fractions 13 and 17. This pattern of size-dependent distribution of mTOR and Raptor is also very similar to that reported by Guan and colleagues [26]. IKKα was found at the highest levels in fractions 13-15. To determine whether IKKα and mTORC2 are associated in these column fractions, IKKα was immunoprecipitated from fractions 13-15 and the immunoprecipitate was analyzed by immunoblotting for IKKα, mTOR, Rictor and Raptor (Figure 5C). The results show that IKKα is associated with mTOR, Rictor and Raptor, with subunits at the highest levels in fraction 14 and 15 and detectable association in fractions 13. Taken together, IKKα associates with both mTORC1 and mTORC2 complexes in PC3 cells.

We previously showed that PI3 kinase (PI3K) induces IKKα-mTORC1 interaction to activate mTORC1 [20-23] and it has been shown by others that mTORC2 activates Akt downstream of PI3K [27]. Therefore, we examined if PI3K induces IKKα-mTORC2 interaction. PC3 cells were mock-treated or treated with different doses of a PI3K inhibitor, LY294002, for one hour and the endogenous IKKα-mTORC2 interaction was examined by co-IP (*via* association with Rictor). The results showed that LY294002 decreases the IKKα-Rictor interaction in a dose dependent manner (Figure 5D). To extend these studies, we determined if Akt activity is required for IKKα-mTORC2 interaction by using perifosine, an Akt inhibitor. Similar to the results generated with the PI3K inhibitor, treatment of PC3 cells with this compound blocked interaction between IKKα and mTORC2 (as measured with Rictor immunoprecipitation; Figure 5E). These data demonstrate that PI3K-Akt signaling promotes IKKα-mTORC2 interaction, functioning in a positive feedforward signaling pathway.

### IKKα induces Akt activity through mTORC2

**Figure 6:**
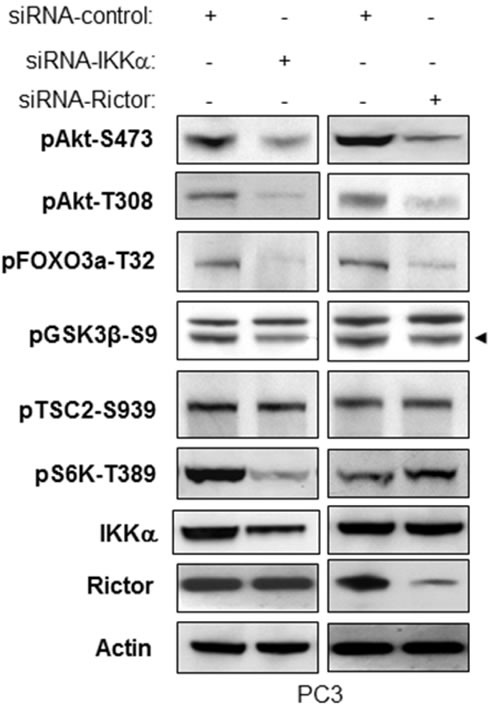
IKKα and Rictor have similar effects on regulation of phosphorylation of Akt and Akt substrates PC3 cells were transfected with control siRNA or siRNA to IKKα or Rictor as indicated. The cells were lysed 48 hrs after transfection and the levels of IKKα, Rictor and β-actin and of endogenous phosphorylation of Akt, FOXO3a, GSK3β (specific band indicated by arrowhead) and S6K were determined by immunoblotting with the indicated antibodies. The experiments were performed three times and a representative blot is shown.

It has been reported that Rictor and mLST8/GβL, both of which are essential components of mTORC2, are required for the hydrophobic motif phosphorylation of Akt/PKB and PKCα, but not S6K1. Moreover, in the mTORC2 complex, SIN1 and Rictor are required only for insulin-induced FOXO3, not for TSC2 or GSK3β phosphorylation following Akt activation [26, 27]. We thus knocked down both Rictor and IKKα in PC3 cells and compared their effects on phosphorylation of Akt, FOXO3a, GSK3β, and S6K. As shown in Figure 6, knockdown of Rictor leads to dramatic decreases of phosphorylation of Akt (both S473 and T308), FOXO3a, and a slight decrease of phosphorylation of GSK3β. However, the phosphorylation of TSC2-Serine 939 and S6K-T389, both of which are indicators of mTORC1 activity, were unchanged. These results are in accordance with the published results by others indicating the involvement of Rictor with mTORC2 but not mTORC1. Consistent with these results, the knockdown of IKKα causes significant reduction of phosphorylation of Akt, FOXO3a, and a slight decrease of phosphorylation of GSK3β without any effect on TSC2 phosphorylation. These results indicate that IKKα loss and Rictor loss show similar results relative to control of mTORC2 activity and function. The difference is that knockdown of IKKα also decreases phosphorylation of S6K-T389, which matches our previous study that IKKα controls mTORC1 activity (Figure 6). These data demonstrated that IKKα positively regulates mTORC2 to Akt activation which is different from its regulation of mTORC1.

### IKKα increases mTORC2 kinase activity directed to Akt at serine 473 but does not affect mTORC2-Rictor interaction

**Figure 7:**
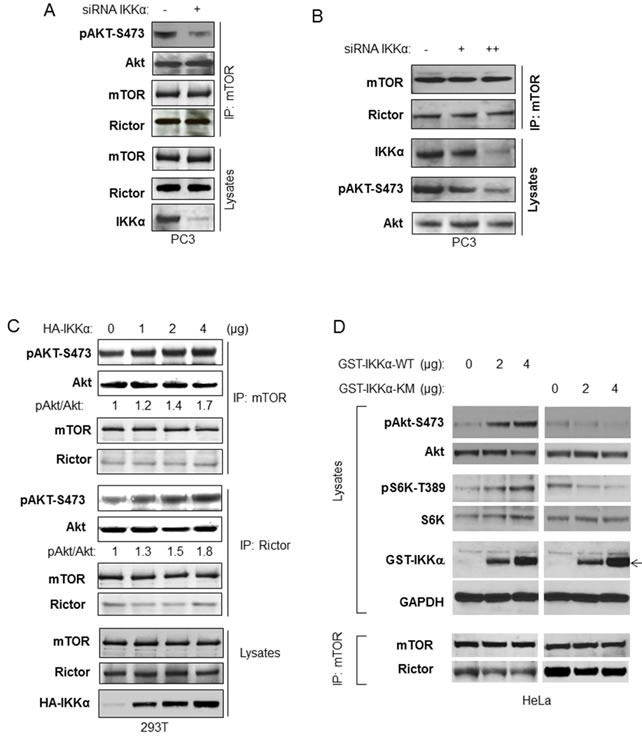
IKKα enhances imTOR/Rictor kinase activity to Akt **A.** PC3 cells were transfected with siRNA IKKα. Endogenous mTOR/Rictor was immunoprecipitated with the mTOR antibody for mTOR/Rictor kinase activity directed toward recombinant Akt. The phosphorylation of Akt (anti-phospho-S473) and total Akt, mTOR and Rictor in the kinase assay system and the amount of mTOR, Rictor and IKKα in the lysate were measured with the indicated antibodies. **B.** PC3 cells were transfected with different amounts of siRNA IKKα. The cells were lyses 48 hours post-transfection and the endogenous mTOR/Rictor was immunoprecipitated with the TOR antibody and incubated with recombinant Akt. The level of mTOR and Rictor in the immunoprecipitates and of endogenous Akt, mTOR, and IKKα in lysates was determined with the indicated antibodies. **C.** HEK293T cells were transfected with different amounts of wild type HA-IKKα, and mTOR and Rictor were immunoprecipitated. mTOR/Rictor kinase activity toward recombinant Akt was determined with phospho-Akt antibody following *in vitro* kinase assay. The protein levels of Akt, mTOR and Rictor in the immunoprecipitates were determined with the indicated antibodies. **D.** HeLa cells were transfected with different amounts of wild type or kinase mutated GST-IKKα (expression indicated by arrow) and the phosphorylation of Akt and S6K, as well as expression of Akt, S6K and GST-IKKα was detected (upper panel). mTOR were immunoprecipitated and levels of mTOR and Rictor in the immunoprecipitate were examined (lower panel).

**Figure 8:**
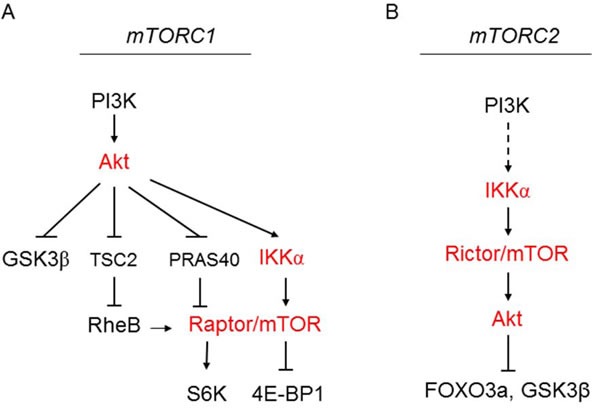
Model for IKKα regulation of mTORC1 downstream of Akt (A) and regulation of mTORC2 upstream of Akt (B) **A.** Shows the downstream effectors of Akt in regulating different key pathways. Akt promotes the interaction of IKKα with the mTORC1 to promote mTORC1 activity. **B.** PI3K signaling *via* Akt promotes the interaction with the mTORC2 complex to promote Akt activity directed to a subset of substrates.

The results described above suggest that IKKα and mTORC2 function to regulate Akt activation in a similar manner, however it was important to determine if IKKα regulates mTORC2 kinase activity directed to Akt phosphorylation. We first tested if IKKα affects mTORC2 kinase activity *in vitro*. mTOR was immunoprecipitated from PC3 from control cells or cells treated with siRNA to IKKα, and the ability of mTOR to phosphorylate recombinant Akt1 was analyzed. The data demonstrate that depletion of IKKα reduces mTOR kinase activity directed to Akt (Figure 7A). Xu et al found that inhibition of IKK reduced the association between Rictor and mTOR, however our studies found that knockdown of IKKα had no effect on the association between Rictor and mTOR (Figure 7A and 7B). We next expressed IKKα in increasing amounts followed by immunoprecipitation of mTOR or Rictor followed by *in vitro* kinase assays using recombinant Akt and anti-phospho S473. Both mTOR immunoprecipitation and Rictor precipitation showed dose-dependent enhancement of Akt S473 phosphorylation following transfection of IKKα (Figure 7C). In order to address whether kinase activity of IKKα is required to promote mTORC2 and the phosphorylation of Akt, we transfected HeLa cells with either wild-type or kinase-inactive IKKα and measured signaling related mTORC1 and mTORC2 activity. The results demonstrate (Figure 7D) that wild-type IKKα stimulates mTORC1 (measured by S6K phosphorylation) and mTORC2 (as measured by the phosphorylation of Akt). However, kinase-inactive IKKα (KM) was not able to activate these pathways. Overall, these data demonstrate that IKKα, through its kinase activity, directly enhances mTORC2 activity directed to phosphorylation of Akt (see model in Figure 8).

## DISCUSSION

Akt and mTOR regulation are intricately linked, with Akt functioning upstream of mTORC1 and mTORC2 regulating Akt activation. mTOR exists in two different complexes, mTORC1 and mTORC2. mTORC1 phosphorylates S6K and 4EBP1 to control cell growth *via* translation, and other substrates for mTORC1 have been identified [2, 7]. Previous studies have demonstrated that Akt activates mTORC1 through the phosphorylation and subsequent inhibition of tuberous sclerosis complex 2 (TSC2), promoting Rheb activation of mTORC1 [7-11]. Furthermore, other publications have shown that Akt phosphorylates PRAS40, a negative regulator of mTORC1 to relieve its inhibitory function on mTORC1 [12, 13]. We previously reported that IKKα is important for efficient activation of mTORC1 activity downstream of Akt-induced signaling [20-23]. In those studies, we found that IKKα interacts with and phosphorylates mTOR in the mTORC1 complex to activate mTORC1, and that Akt signaling drives the IKKα-mTORC1 interaction. Interestingly, the canonical IKK complex (IKKα, IKKβ, IKKγ) was recruited into the mTORC1 complex, yet IKKβ was found not to be involved in mTORC1 activation but was shown to be important for a reciprocal activation of NF-κB controlled by mTORC1 [23]. Another report found that IKKβ can phosphorylate TSC1 in response to TNF to promote mTORC1 activation [28], but our studies indicate that IKKα is the dominant form of IKK downstream of Akt signaling [21].

In the current study, we show that IKKα associates with the mTORC2 complex and positively regulates mTORC2 kinase activity directed to Akt (Figure 8). Others [24] have suggested that IKKβ is important in this response and that IKK promotes Rictor association with mTOR in the mTORC2 complex, but our data (at least in the cancer cells studied) indicate that IKKα is more important than IKKβ in controlling Akt activation and that there is no reduction in Rictor-mTOR association following IKKα knockdown. Data from our studies indicate that PI3K and Akt drive IKKα onto the mTORC2 complex, promoting a feedforward process to maintain mTORC2 activity. Previously we found that the IKKα-directed phosphorylation (S1415) of mTOR in the mTORC1 complex stimulates mTORC1 kinase activity [23]. While expression of an S1415A mTOR mutant suppressed mTORC1 activity, it did not suppress phosphorylation of Akt [23], suggesting that phosphorylation at this site by IKKα does not regulate mTORC2 activity. Yet our data indicate that the kinase activity of IKKα is required to promote mTORC2 and Akt activation (Figure 7D). Thus a mechanism to explain the effect of IKKα on promoting mTORC2 activity is not known. Our findings provide insights into a previous report showing that the activation of Akt downstream of BAFF receptor signaling requires IKKα [29].

Knockout of Rictor in mice demonstrated that mTORC2 activates Akt (phosphorylation at S473 and T308) and controls certain, but not all, Akt substrates [27]. The animal study reported by Sabatini group demonstrated that both Rictor and mLST8 (GβL) are involved in mTORC2-induced phosphorylation of FOXO3a, but not TSC2 or GSK3β. In 2006, mSin1 was identified as another component of mTORC2 but not mTORC1 [26, 30]. It has been demonstrated that mSin1 is necessary for the assembly of mTORC2 and for its capacity to phosphorylate Akt/PKB [26, 30]. In addition, similar to the function of Rictor to mTORC2, mSin1 mediates Akt phosphorylation of FOXO3a [30], while other Akt targets TSC2, GSK3, and the TORC1 effectors, S6K and 4E-BP1 were unaffected. A mechanism to explain the selective phosphorylation of Akt substrates in these studies is not understood. Our data indicate that IKKα acts as an upstream regulator of mTORC2 to control mTORC2 activity to Akt and its substrate FOXO3a, which is similar to the function of Rictor and Sin1 in regulating Akt and its downstream targets. Others have reported that IKK-related kinases IKKε and TBK1 can directly phosphorylate Akt at S473 [31, 32], thus our data indicate an additional pathway whereby IKK family members can drive Akt activation. Our observations that IKKα is critical for regulation of both mTORC1 and mTORC2, along with its established roles in the activation of NF-κB signaling, indicate IKKα as an important cancer therapeutic target.

## MATERIALS AND METHODS

### Cell lines, cell culture and transfection

IKK wild-type and IKKα^−/−^ were provided by I. Verma and M. Karin. The prostate cancer cell lines PC3, LNCaP and DU145, HEK293T, Hela, A549, PANC1 and MiaPaCa-2 cell lines were from ATCC. The LNCaP cell line was cultured in PMRI1640 with 10% fetal bovine serum (FBS), 2 mM glutamine, and 100 U/ml penicillin and streptomycin (GIBCO). All other cells were maintained in Dulbecco's modified Eagle's medium (DMEM) supplemented with 10% fetal bovine serum (FBS), 2 mM glutamine, and 100 U/ml penicillin and streptomycin (GIBCO). Transfections were performed using Lipofectamine and Plus (Invitrogen) following the manufacturer's instructions. In brief, 3-4 hours after transfection, cells were recovered in full serum for 36 hours or in full serum for 24 hr and then serum-starved for 16 to 24 hours as indicated.

### Antibodies and reagents

Antibodies were obtained from the following sources. Antibodies against IKKα, IKKβ, and mTOR were obtained from Upstate Biotechnology. Raptor and Rictor antibodies were obtained from Bethyl Laboratories. Anti-HA and anti-Flag antibodies were obtained from Roche and Sigma, respectively. Anti-Actin was obtained from Calbiochem. The anti-S6K and control rabbit IgG, as well as HRP-labeled anti-mouse and anti-rabbit secondary antibodies were from Santa Cruz Biotechnology. Recombinant Human inactive Akt is from Upstate Biotechnology. All other antibodies were from Cell Signaling. Other reagents were obtained from the following sources: Insulin was from Invitrogen Corporation. Protease and phosphatase inhibitor cocktails were from Roche. The CHAPS was from Pierce. Protein A and protein G agarose beads were from Invitrogen Life Technologies.

### RNA interference

siRNA SMARTpool IKKα (Catalog #M-003473) employed in all experiments except Figure 1B. Other two siRNA, labeled as siRNA-IKKα-2 and siRNA-IKKα-3 in Figure 1B are from Santa Cruz and Sigma, respectively. Raptor and Rictor were from Dharmacon. Each of these represents four pooled SMART selected siRNA duplexes that target the indicated gene. HeLa, PC3, LNCaP and DU145 cell lines cells were transfected with indicated SMARTpool siRNA or nonspecific control pool using DharmaFECT 1 reagent (Dharmacon) according to the manufacturer's instructions. In brief, 20 nM final concentration of siRNA was used to transfect cells at 60%-70% confluency. Twenty-four hours after transfection, cells were recovered in full serum or were serum-starved 16 hr before harvest. Cells were harvested 48 to 72 hr after siRNA transfection.

### Cell lysis, immunoblotting and coimmunoprecipitation

In brief, cells growing in 100 mm dishes were rinsed twice with cold PBS and then lysed on ice for 20 min in 1 ml lysis buffer (40 mM Hepes pH 7.5, 120 mM NaCl, 1 mM EDTA, 10 mM pyrophosphate, 10 mM glycerophosphate, 50 mM NaF, 0.5 mM orthovanadate, and EDTA-free protease inhibitors (Roche)) containing 1% Triton X-100. After centrifugation at 13,000Xg for 10 min, samples containing 20-50 μg of protein were resolved by SDS-PAGE and proteins transferred to Pure Nitrocellulose Membrane (Bio-Rad Lab.), blocked in 5% nonfat milk, and blotted with the indicated antibodies. For immunoprecipitation experiments, the lysis buffer contained 0.3% CHAPS instead of 1% Triton. 4 μg of the indicated antibodies were added to the cleared cellular lysates and incubated with rotation for 6-16 hours. Then 25 μl of protein G agorose was added and the incubation continued for 1 h. Immunoprecipitates captured with protein G-agorose were washed three times with the CHAPS Lysis Buffer, two times by wash buffer A (50 mM Hepes, PH 7.5, 150mM NaCl), and boiled in 4x SDS samples buffer for western blot.

### *In vitro* mTOR/Rictor kinase assay

We followed the protocol for the *in vitro* assay of Rictor/mTOR kinase acivity directed to Akt [4]. In brief, transfected HEK293T cells were grown in 100mm dishes for 48 hours in DMEM containing 10% FBS, and lysed in 1ml lysis buffer with 0.3% CHAPS. Half of total cell lysate was incubated with anti-mTOR or anti-Rictor antibody for 3 hours, followed by another hour of incubation with 25 μl protein G agarose beads. Immunoprecipitates were washed four times by lysis buffer, once by the Rictor-mTOR kinase buffer (25 mM HEpes PH 7.5, 100 mM potassium acetate, 1 mM MgCl2). For kinase reaction immunoprecipitates were incubated in a final volume of 15 μl for 20 min at 37°C in the rictor-mTOR kinase buffer containing 500 ng inactive Akt1/PKB1 (Akt1/PKB1, Upstate Biotechnology, #14-279) and 500 μM ATP. The reaction was stopped by the addition of 200 μl ice-cold Enzyme Dilution buffer (20 mM MOPS, pH 7.0, 1 mM EDTA, 0.01% Brij 35, 5% glycerol, 0.1 % 2-mercaptoethanol, 1 mg/ml BSA). After a quick spin, the supernatant was removed from the protein G-sepharose and analyzed by immunoblotting.

### Statistics

Data from the *in vitro* experiments are expressed as mean ± SEM from a minimum of 3 independent experiments. Comparison between groups were carried out by 2-way ANOVA or Student's *t* test, and a *P* value of less than 0.05 was considered significant.
